# A Simple Method for DNA Extraction from Mature Date Palm Leaves: Impact of Sand Grinding and Composition of Lysis Buffer

**DOI:** 10.3390/ijms11093149

**Published:** 2010-09-08

**Authors:** Ibrahim A. Arif, Mohammad A. Bakir, Haseeb A. Khan, Anis Ahamed, Ahmad H. Al Farhan, Ali A. Al Homaidan, Mohammad Al Sadoon, Ali H. Bahkali, Mohammad Shobrak

**Affiliations:** Molecular Fingerprinting and Biodiversity Unit, Prince Sultan Research Chair for Environment and Wildlife, College of Sciences, King Saud University, Riyadh, Saudi Arabia; E-Mails: iaarif@hotmail.com; (I.A.A.); mabakir@yahoo.com (M.A.B.); nanisahamed@rediffmail.com (A.A.); alfarhan@ksu.edu.sa (A.H.A.F.); homaidan@ksu.edu.sa (A.A.A.H.); msadoon@ksu.edu.sa (M.A.S.); abahkali@ksu.edu.sa (A.H.B.); mshobrak@gmail.com (M.S.)

**Keywords:** DNA extraction, PCR, plants, tough leaves, date palm, Phoenix dactylifera

## Abstract

Molecular marker techniques have been widely used for cultivar identification of inbred date palms (*Phoenix dactylifera* L.; Arecaceae) and biodiversity conservation. Isolation of highly pure DNA is the prerequisite for PCR amplification and subsequent use such as DNA fingerprinting and sequencing of genes that have recently been developed for barcoding. To avoid problems related to the preservation and use of liquid nitrogen, we examined sterile sand for grinding the date palm leaves. Individual and combined effects of sodium chloride (NaCl), polyvinylpyrrolidone (PVP) and lithium chloride (LiCl) with the cetyltrimethylammonium bromide (CTAB) method for a DNA yield of sufficient purity and PCR amplification were evaluated in this study. Presence of LiCl and PVP alone or together in the lysis buffer did not significantly improve the DNA yield and purity compared with the addition of NaCl. Our study suggested that grinding of date palm leaf with sterile sand and inclusion of NaCl (1.4 M) in the lysis buffer without the costly use of liquid nitrogen, PVP and LiCl, provides a DNA yield of sufficient purity, suitable for PCR amplification.

## 1. Introduction

Most of the plant DNA isolation methods including commercial kits require grinding of the plant material in liquid nitrogen. By virtue of this, any tissue immersed in liquid nitrogen instantly becomes brittle solid to facilitate crushing into powder, with an additional advantage of maintaining the tissue at low temperature. However, the grinding step in liquid nitrogen may be omitted for soft, easy-to-grind materials such as flower-petals [1]. Date palm (*Phoenix dactylifera* L.; Arecaceae), a long-lived dioecious monocotyledon, plays an important socioeconomic role in the Middle East. Date palm leaves are hard, fibrous and the extraction of genomic DNA from the leaves is difficult. To avoid the problems related with the preservation and use of liquid nitrogen, acid-washed sand or glass powder were used for grinding the leaves of date palm [2]. DNA has also been extracted using sand from many genera of rain forest plant species [3]. In many small laboratories of developing countries, liquid nitrogen is not always available. Storage and maintenance of liquid nitrogen is also difficult. The highly versatile cetyl trimethylammonium bromide (CTAB) method has been used for the extraction of DNA from various plant materials [4].

There are three main contaminants associated with plant DNA that can cause considerable difficulties when conducting PCR experiments: polyphenolic compounds, polysaccharides and RNA. Presence of phenolic pool like quercetin, isorhamnetin heterosides, (+)-catechin, (−)-epicatechin, 5-caffeoylshikimic acid (dactylifric acid) and its positional isomers (3-caffeoylshikimic acid and 4-caffeoylshikimic acid) that are present in the leaves of date palm [5] may interfere with the successful isolation of PCR amplifiable DNA. Inclusion of sodium chloride (NaCl) with the lysis buffer has been used for removing polysaccharides [6]. Likewise, polyvinylpyrrolidone (PVP) has been recommended for removal of polyphenolic compounds [7] and lithium chloride (LiCl) for RNA [3].

Recently, a combination of NaCl, PVP and LiCl has been used with the CTAB method for the isolation of genomic DNA from coniferous tissues (*Taxus baccata*) [8]. However, the individual effects of NaCl, PVP and LiCl as well as their typical combinations have not been tested for optimal isolation of genomic DNA from plant tissues. In this study, we have examined the individual and combined effects of NaCl, PVP and LiCl in conjunction with the basic CTAB protocol. Our main objective was to optimize a simple, inexpensive and rapid procedure for DNA isolation from tough leaves (date palm) without compromising the yield and purity of DNA.

## 2. Materials and Methods

### 2.1. DNA Extraction

Fresh leaf of date palm (100 mg) was placed in a sterile mortar. Sterile sand (50 mg) and 500 μL of lysis buffer (Table 1; lysis buffers A to E) were added separately to the sterile mortar. Leaf sample was finely crushed using mortar and pestle and allowed to dry at room temperature for about 5 min. Crushed leaf sample with sand (100 mg) was transferred into a 1.5 mL eppendorf tube. The same lysis buffer (1,000 μL) that was used for grinding the leaf was added to the tube and vortexed briefly. The tube was then kept in a water bath at 60 °C for 30 min. After mixing by brief vortex, the tube was centrifuged at 9,500 g for 5 min. An aliquot of supernatant (200 μL) was transferred to a new tube, taking care to avoid carryover of any dirt or debris. An equal volume (200 μL) of chloroform: isoamyl alcohol (24:1) was added and the tube was shaken gently top to bottom for 5 min followed by centrifugation at 9,500 g for 5 min. The supernatant (200 μL) was transferred to a new tube and sodium acetate (3.0 M; 20 μL) plus cold isopropanol (500 μL) were added gently and the tube was kept in the freezer for 5 min followed by centrifugation at 11,500 g for 10 min. The resulting supernatant was discarded and 500 μL of 70% cold ethanol was added and vortexed briefly. After centrifugation at 7,000 g for 5 min, the supernatant was discarded and the tube contents were air dried at room temperature. DNA was eluted with 100 μL of TE buffer and kept at 4 °C for further use. Experiments were conducted with 4 individual replicates.

### 2.2. DNA Quantification

The purity and quantity of isolated DNA were determined spectrophotometrically (GeneQuant-1300; GE Healthcare, UK). Optical density (OD) values at 230, 260 and 280 nm were recorded.

### 2.3. RAPD-PCR Analysis of Isolated DNA

Ready-To-Go RAPD analysis beads (GE Healthcare, UK) were used for RAPD-PCR analysis. PCR reaction mixture of 25 μL contained a single bead, 25 pmol of a single RAPD primer, 100 ng of template DNA and sterile distilled water. The bead contained thermostable polymerase (AmpliTaq™ DNA polymerase and stoffel fragment, dNTPs (0.4 mM each), BSA (2.5 μg) and buffer [3 mM MgCl_2_, 30 mM KCl and 10 mM Tris, (pH 8.3)]. The primer used in this study was a 10-mer of arbitrary sequence (5′-GTTTCGCTCC-3′; GE Healthcare, UK).

PCR reaction was performed using a Veriti thermal cycler (Applied Biosystems). PCR condition included 1 cycle of 95 °C for 5 min, followed by 45 cycles of 95 °C for 1 min, 36 °C for 1 min and 72 °C for 2 min. A long (20 × 14 cm) 1.5% agarose gel using 1x TBE buffer containing 0.5 μg/mL of ethidium bromide was used for electrophoresis purposes. Gel image was visualized using Proxima C16 Phi+ (Isogen Life Science) UV transluminator and Opticom (version 3.2.5, OptiGo) imaging system. Gel image analysis and the sizes of RAPD bands were determined using 100 base-pair ladder (GE Healthcare) and TotalLab (TL100 1D; version 2008.01) software. Only amplicons that occurred in all replicate sample amplifications were used in the analysis.

### 2.4. Data Analysis

OD values of DNA extracted by different lysis buffers were analyzed by ANOVA. Amplified fragments of RAPD-PCR were scored as present (1) or absent (0). Only clear and major bands were scored [9]. Pairwise comparisons based on the proportion of shared bands and bootstrap values (1000 replications) were calculated using the program Free-Tree [10].

## 3. Results and Discussion

### 3.1. DNA Yield and Purity

**Figure 1:**
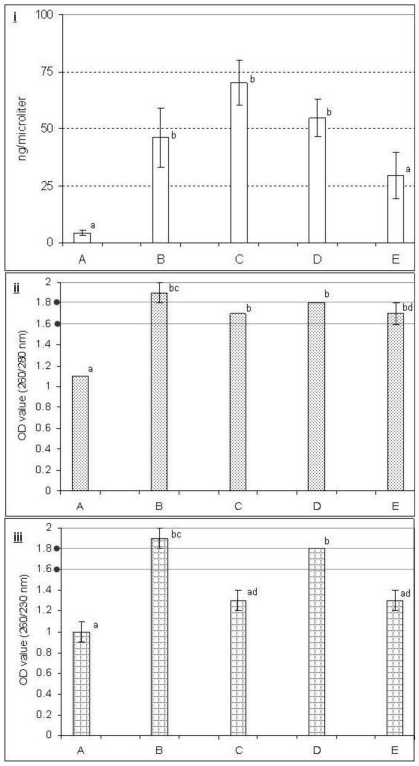
Effect of different lysis buffers (**A**, **B**, **C**, **D** and **E**) on DNA yield (**i**) and purity (**ii** and **iii**) from mature date palm leaf. Bar represents standard error of the mean. OD, Optical density; * Yield, *F* = 7.14, *P* = 0.002; ** OD value (260/280 nm), *F* = 41.54; *P* = 0; *** OD value (260/230 nm), *F* = 15.77, *P* = 0; Values expressed by different letters on the bars are significantly different (Tukey test, 5% level).

Effects of different buffers on DNA yield and purity are illustrated in Figure 1. The results showed that different buffers that we examined for the extraction of DNA provided significantly different levels of yield and purity. DNA extracted with the buffers B, C and D produced a higher yield compared with buffers A and E (Figure 1, upper panel). The ratio of ODs at 260 nm and 280 nm is commonly used to assess the purity of DNA with respect to protein contamination, since proteins (in particular, the aromatic amino acids) tend to absorb at 280 nm. The method dates back to 1942, when Warburg and Christian showed that this ratio is a good indicator of nucleic acid contamination in protein preparations [11]. A ratio of ~1.8 is generally accepted as pure DNA. If the ratio is appreciably lower, it may indicate the presence of protein, phenol or other contaminants that absorb strongly at or near 280 nm. We observed that the ratio of OD values at 260/280 nm were more or less similar (1.7 to 1.9) for all the buffers except buffer A (Figure 1, middle panel). A secondary measure of nucleic acid purity is based on the ratio of OD values at 260 nm and 230 nm. The 260/230 values for pure nucleic acid are often higher than the respective 260/280 values. Expected 260/230 values are commonly in the range of 2.0–2.2. If the ratio is appreciably lower than expected, it may indicate the presence of contaminants which absorb at 230 nm. Ethylenediaminetetraacetic acid (EDTA), carbohydrates and phenol absorb near 230 nm. We found that OD values of 260/230 nm for DNA-extraction using buffers B and D (1.8 to 1.9) were significantly different compared with the others (Figure 1, lower panel).

### 3.2. PCR Amplification

**Figure 2:**
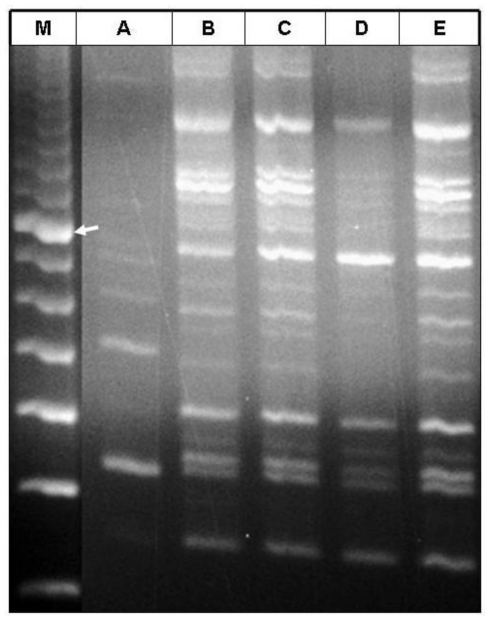
RAPD-PCR product profiles of extracted DNA from mature leaves of date palm using different lysis buffers. Lane M, 100 bp molecular weight marker; lanes A to E, different lysis buffers. Arrow indicates the 800 bp position.

**Figure 3:**
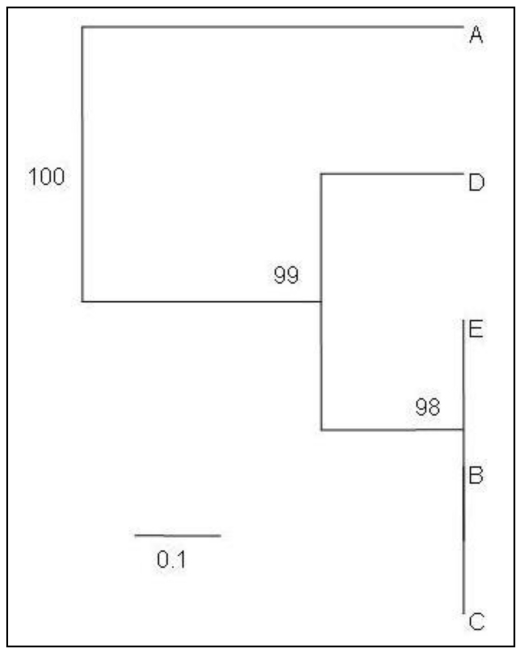
UPGMA tree constructed using the Jaccard method showing the relationships among isolated DNA using different lysis buffers based on RAPD profiles. Bootstrap values (expressed as percentages of 1000 replications) >50% are shown at branch points.

RAPD-PCR was conducted to examine the amplification of the isolated DNA by different lysis buffers. DNA isolated by lysis buffers B, C and E showed satisfactory amplifications in PCR. The fingerprint we obtained by using the DNA extracted by these buffers provided higher resolution than those using buffers A and D which did not result in the expected PCR products (Figures 2 and 3). The UPGMA tree constructed using the Jaccard method form the RAPD fingerprinting profile placed buffer B, C and E in the same cluster and separated them from buffers A and D (Figure 3). DNA isolated by using the lysis buffers B, C and E produced 9 clear bands whereas buffers A and D produced 2 (509 and 327 bp) and 6 (1225, 725, 400, 336, 327 and 243 bp) bands, respectively (Figure 2). DNA isolated with buffers B, C and E shared the band of 1225, 986, 937, 894, 725, 400, 336, 327 and 243 bp. Therefore, these seem to be the typical fingerprinting bands of date palm produced by the primer and PCR-conditions used for the experiment. DNA extracted with buffer D was lacking band 986, 937 and 894 bp bands. DNA isolated with buffer A shared only one band (327 bp) with the other lysis buffers used in this study.

### 3.3. Effect of PVP, LiCl and NaCl

PVP has long been used to bind the polyphenolic compounds and LiCl for removing RNA [12]. In general, PVP is used to purge polyphenols [7] and may promote precipitation of the phenolic compounds [13,14]. PVP forms complex hydrogen bonds with polyphenolic compounds which can be separated from DNA by centrifugation [7]. The presence of polyphenolic compounds was observed to be reduced by using PVP in the DNA extraction procedure [15]. However, our study showed that inclusion of PVP (buffer C) in the lysis buffer did not significantly improve the DNA yield or purity compared with NaCl alone (buffer B) (Figure 1). However, RAPD profiles were similar for both the buffers B and C (Figures 2 and 3).

In order to eliminate RNA from the extracts, LiCl has been used in lysis buffers to selectively precipitate the large molecules of RNA. This selective precipitation is more advantageous than RNAase treatment, in which the RNA is enzymatically degraded into smaller units, but not removed from the extracts [3,16,17]. Our study showed that inclusion of LiCl (buffer D) did not differ from inclusion of NaCl alone (buffer B) in terms of DNA yield and purity (Figure 1) and RAPD fingerprinting results also showed that inclusion of LiCl (buffer D) in the lysis buffer provided less resolution than inclusion of NaCl alone (buffer B) (Figures 2 and 3). Combination of PVP and LiCl (buffer E) in the lysis buffer produced less DNA yield and purity (Figure 1). However, RAPD results were similar in buffers B and C (Figures 2 and 3). Among the contaminants, polysaccharides are difficult to separate from DNA [18]. Polysaccharides interfere with several biological enzymes such as polymerases, ligases and restriction endonucleases [19,20] and the removal of polymerase inhibitors such as polysaccharides favors DNA amplification by PCR [6,15,21]. However, several polymerases for PCR have hit the market in the last decade with typical advantages including robustness against all kinds of inhibitors. Further studies are warranted to evaluate these polymerases for PCR amplification of plant DNA.

This study showed that the addition of higher molar concentration of NaCl (1.4 M) alone (buffer B) in the lysis buffer provided better yield and purity compared with the addition of PVP and LiCl (buffers C, D and E) (Figure 1). Thus, the presence of NaCl in the lysis buffer seems to play an important role for the yield of DNA, purity and PCR amplification as buffer A (without addition of NaCl) failed to produce higher yield, purity and RAPD-PCR fingerprinting resolution (Figures 1–3). Inclusion of NaCl alone in the lysis buffer provided significantly better results compared to the addition of LiCl and PVP (Figure 1). High molar concentration of NaCl inhibits co-precipitation of the polysaccharides and DNA [12]. Most of the polysaccharides remove effectively in a single high-salt precipitation at 1.0–2.5 M NaCl. However, at very high concentrations, such as 3.0 M NaCl, the salt precipitates out of solution. NaCl (1.0 M) facilitates the removal of polysaccharides by increasing their solubility in ethanol so that they did not co-precipitate with the DNA [6]. However, higher concentrations of NaCl (more than 2.5 M) were found to be more effective [15]. Presence of LiCl and PVP alone or together in the buffer did not improve the DNA yield and purity compared with the addition of NaCl alone.

## 4. Conclusion

In conclusion, this study suggests that grinding of date palm leaves with sterile sand and inclusion of NaCl (1.4 M) in the lysis buffer without the costly use of liquid nitrogen, PVP and LiCl, provides a DNA yield of sufficient purity, suitable for PCR amplification and subsequent use.

## Figures and Tables

**Table 1:** Constituents of lysis buffers (100 mL, pH 8.0).

Lysis Buffer	Main components	Additives	
A	Trizma (1.21 g) + Na_2_EDTA (0.4 g) + CTAB (2.0 g)	-	-
B	Trizma (1.21 g) + Na_2_EDTA (0.4 g) + CTAB (2.0 g)	NaCl (8.12 g)	-
C	Trizma (1.21 g) + Na_2_EDTA (0.4 g) + CTAB (2.0 g)	NaCl (8.12 g)	PVP ( 2.0 g)
D	Trizma (1.21 g) + Na_2_EDTA (0.4 g) + CTAB (2.0 g)	NaCl (8.12 g)	LiCl ( 0.2 g)
E	Trizma (1.21 g) + Na_2_EDTA (0.4 g) + CTAB (2.0 g)	NaCl (8.12 g)	PVP (2.0 g) + LiCl (0.2 g)

Trizma (Sigma-Aldrich), 2-amino-2-hydroxymethyl-1,3-propandiol [Tris(hydroxymethyl) aminomethane; Na_2_EDTA, ethylinediamine-N,N,N^2^,N^2^-tetra-acetic acid disodium salt; CTAB, cetyl trimethylammonium bromide; NaCl, sodium chloride; PVP, polyvinylpyrrolidone; LiCl, lithium chloride; -, no additives.

## References

[1] Lin JZ Ritland K Flower petals allow simpler and better isolation of DNA for plant RAPD analysis Plant Mol. Biol. Rep 1995 13 210 213

[2] Ouenzar B Hartmann C Rode A Benslimane A Date Palm DNA mini-preparation without liquid nitrogen Plant Mol. Biol. Rep 1998 16 263 269

[3] Scott KD Playford J DNA lysis technique for PCR in rain forest plant species Biotechniques 1996 20 974 978 8780866 10.2144/96206bm07

[4] Doyle JJ Doyle JL Isolation of plant DNA from fresh tissue Focus 1990 12 13 15

[5] Ziouti A El-Modafar C Fleuriet A El-Boustani S Macheix JJ Phenolic compounds in date palm cultivars sensitive and resistant to *Fusarium oxysporum* Biol. Plantarum 1996 38 451 457

[6] Fang G Hammar S Rebecca R A quick and inexpensive method for removing polysaccharides from plant genomic DNA Biotechniques 1992 13 52 56 1503775

[7] Maliyakal EJ An efficient method for isolation of RNA and DNA from plants containing polyphenolics Nucleic Acids Res 1992 20 2381 1375739 10.1093/nar/20.9.2381 PMC312364

[8] Barzegari A Vahed SZ Atashpaz S Khani S Omidi Y Rapid and simple methodology for isolation of high quality genomic DNA from coniferous tissues (*Taxus baccata*) Mol. Biol. Rep 2010 37 833 837 19649730 10.1007/s11033-009-9634-z

[9] Collard BCY Mackill DJ Start codon targeted (SCoT) polymorphism: a simple, novel DNA marker technique for generating gene targeted markers in plants Plant Mol. Biol 2009 27 86 93

[10] Pavlícek A Hrdá S Flegr J Free-Tree--freeware program for construction of phylogenetic trees on the basis of distance data and bootstrap/jackknife analysis of the tree robustness. Application in the RAPD analysis of genus *Frenkelia* Folia. Biol 1999 45 97 99 10730897

[11] Sambrook J Russell DW Molecular Cloning: A Laboratory Manual3rd ed Cold Spring Harbor Laboratory Press New York, NY, USA 2001

[12] Jobes DV Hurley DL Thien LB Plant DNA isolation: A method to efficiently remove polyphenolics, polysaccharides, and RNA Taxon 1995 44 379 386

[13] Kim CS Lee CH Shin JS Chung YS Hyung NI A simple and rapid method for isolation of high quality genomic DNA from fruit trees and conifers using PVP Nucleic Acids Res 1997 25 1085 1086 9023124 10.1093/nar/25.5.1085 PMC146538

[14] Khanuja SPS Shasany AK Darokar MP Kumar S Rapid isolation of DNA from dry and fresh samples of plants producing large amounts of secondary metabolites and essential oils Plant Mol. Biol. Rep 1999 17 1 7

[15] Lodhi MA Ye GN Weeden NF Reisch BI A simple and efficient method for DNA lysis from grapevine cultivars, *Vitis* species and *Ampelopsis* Plant Mol. Biol. Rep 1994 12 6 13

[16] Ostrowska E Muralitharan M Chandler S Volker P Hetherington S Dunshea F Optimizing conditions for DNA isolation from *Pinus radiate* In Vitro Cell Dev. Biol Plant 1998 34 108 111

[17] Ribeiro RA Lovato MB Comparative analysis of different DNA lysis protocols in fresh and herbarium specimens of the genus *Dalbergia* Genet. Mol. Res 2007 6 173 187 17469067

[18] Murray MG Thompson WF Rapid isolation of high molecular weight DNA Nucleic Acids Res 1980 8 4321 4325 7433111 10.1093/nar/8.19.4321 PMC324241

[19] Shioda M Muofushi KM Selective inhibition of DNA polymerase by a polysaccharide purified from slime of *Physarum polycephalum* Biochem. Biophys. Res. Commun 1987 146 61 66 3606625 10.1016/0006-291x(87)90690-5

[20] Richards E Ausubel FM Kingston RE Moore DD Smith JA Seidman JG Struhl K Preparation of genomic DNA from plant tissue Current Protocols in Molecular Biology Greene Publishing Associates and Wiley-Interscience New York, NY, USA 1988

[21] Webb DM Knapp SJ DNA lysis from a previously recalcitrant plant genus Plant Mol. Biol. Rep 1990 8 180 185

